# Editorial: The Role of Thyroid Hormones in Vertebrate Development

**DOI:** 10.3389/fendo.2019.00863

**Published:** 2019-12-12

**Authors:** Laurent M. Sachs, Marco A. Campinho

**Affiliations:** ^1^Département Adaptation du Vivant, UMR 7221 CNRS, Muséum National d'Histoire Naturelle, Paris, France; ^2^Centre of Marine Sciences, Faro, Portugal

**Keywords:** thyroid hormones, development, vertebrates, teleosts, anurans, birds, mammals

In vertebrates, thyroid hormones (TH) have long been recognized as essential factors in vertebrate natal and postnatal development. Since TH role was first suggested in the Eighteenth century when a relationship between goiter and cretinism was found, TH was shown to play key roles in development of the nervous system in vertebrates, controlling diverse processes such as neurogenesis, cell migration, apoptosis, differentiation, and maturation. Furthermore, the role of TH is more diversified. TH act on several other key events during development including morphogenesis and metabolism. Notwithstanding, research into the role of TH in vertebrate development is still lagging. This is mainly due to the relatively easy by which thyroid/TH linked pathologies could be treated “simply” by TH supplementation and also the pleiotropic nature of TH action that makes it difficult to identify and study discrete roles of TH in various aspect of development. However, for the patient, the therapeutic interventions are not always satisfactory, with the conservation of adverse effects on the quality of life. In this special issue of Frontiers in Endocrinology, we aim to address state of the art on TH role in vertebrate development, with a comparative perspective from teleosts to humans to highlight the huge conservation of the TH signaling pathway in vertebrates. The biology of TH is complex with highly regulated production of the TH in the gland, entry, activation, and binding of the TH to cognate receptors in target cells. Actually, new scientific advances and technologies are now providing tools to dissect the developmental role of TH on vertebrate development, both embryonic and post-natal. Given that TH constitute integrative physiological signals in vertebrates a new scenario is emerging whereas TH seem to be important in the development and maturation of most organ systems in vertebrates during their different life transitions.

The manuscripts published highlight the evolutionary conserved role of TH on vertebrate natal and post-natal neurodevelopment from fishes, anurans, birds to humans. First, Rabah et al. review into the role of blood and cerebrospinal fluid TH transporter proteins in vertebrate development with special incidence in transthyretin (TTR) in humans and associated pathologies. The original localization of TTR in the cerebrospinal fluid open new perspectives on the biological understanding of TH function and TH availability in brain formation. In the same register, Stepien and Huttner review the current knowledge about TH delivery (transport), conversions (metabolism), and function in the developing mammalian brain, including neurogenesis and brain maturation. They also discuss their potential role of TH in vertebrate brain evolution and offer future directions for research.

Next, thanks to the contribution of non-mammalian models to dissect the developmental role of TH on vertebrate development, both embryonic and post-natal. The post-natal role of TH in vertebrate development is long known from anuran studies. Amphibian metamorphosis is a valuable model to study postembryonic development in vertebrates because this developmental process is independent of maternal influence and avoids the difficulty to manipulate the uterus-enclosed embryos and neonates. Interestingly, TH is not the only endocrine signal involved in frog metamorphosis. As reported by Sachs and Buchholz, if TH is probably the most important hormone, glucocorticoids (GCs) can modulate the rate of developmental progress induced by TH and may also have direct actions required for completion of metamorphosis independent of their effects on TH signaling. Here, they provide a new review and analysis of the crosstalk between the two endocrine pathways that are strongly conserved during vertebrate evolution. Next, coming back on the effect of TH on brain development, Wen et al. were able using an inactivating mutation in the gene that encodes TH receptor alpha (TRα) to provide evidences that TRα is required for TH-dependent neural cell proliferation during tadpole metamorphosis, a suggested for mammals. Indeed, TRα accounts for 95% of the gene regulation responses to TH. Another pertinent use of amphibian model is the understanding of TH action on the most notable phenomena in developmental biology resulting in morphological changes: the limb development and growth along with tail resorption. These two phenomena are linked to locomotive switch. Yaoita highlights how this switch require elaborate regulation with differential TH sensitivity of the tail and hindlimbs. He also reviews the mechanism leading to tail resorption that occurs through two mechanisms, suicide and murder.

Moreover, today the access to transcriptome analysis in several Anuran species strongly improve capacity to distinguish between species specific transcriptional program and shared program. Wang et al. findings suggest that tail resorption in tow *Microhyla fissipes* and *Xenopus laevis* shares many programs. These results help to reveal important mechanistic insights governing mammalian postembryonic developments, with strong impact to understand the life transition from an aquatic environment to an air breathing environment. Another example comes from an Anuran with a different life cycle than the classical model, the Xenopus. Indeed, although frogs present indirect development with larval phase and metamorphosis other frogs have direct development and bypass tadpole stage. It is remarkable that the developmental action of TH is highly conserved. Laslo et al. show that, in comparison to biphasic anuran species, in the direct-developing frog *Eleutherodactylus coqui*, TH also promotes limb development and the same TH-signaling genes found in Xenopus are involved. Remarkably, limb development occurs before thyroid gland development highlighting the critical role of maternal TH in these anurans, critical role that has also been reported in mammals. Amphibian clade provides another group of model organism in biomedical research. Urodela, such as *Ambystoma mexicanum* (Axolotl) has amazing ability to regenerate after injury and ability to retain juvenile morphology into the adult phase of life. The axolotl does not typically undergo a metamorphosis, but TH can induce transformation. Crowner et al. discuss paedomorphic development and metamorphosis of axolotl. This anuran constitutes a formidable example of how different regulation of TH signaling during metamorphosis can give rise to singular developmental outcomes in vertebrates. With the recent completion of the axolotl genome assembly and established methods to manipulate gene functions, the axolotl is poised to provide new insights about paedomorphosis and the role of thyroid hormone in development and evolution. Using the same model and a combination of developmental endocrinology, functional genomics, and network biology to compare the transcriptional response of tailfin to TH, in the post-hatching paedormorphic axolotl and *Xenopus* tadpoles, Kerdivel et al. first show that axolotl tailfin undergoes a TH-dependent transcriptional response at post embryonic transition, despite the lack of visible anatomical changes and second propose that a subnetwork of cellular sensors and regulators, display opposite transcriptional programs conducting alternative tailfin fate (maintenance vs. resorption)post-hatching.

Today, amphibians are not the only non-mammalian models to dissect the developmental role of TH on vertebrate development. In most teleosts, metamorphosis encompasses a dramatic post-natal developmental process where the free-swimming larvae become a fully formed juvenile fish (Campinho). In the flatfishes, the morphological changes are more dramatic with the migration of one eye to the opposite side of the head while simultaneously the symmetric pelagic larva develops into an asymmetric benthic juvenile. Thus, flatfish metamorphosis is a remarkable example of the capacity of TH-signaling in shaping adaptation and evolution. Only recently, the powerful Zebrafish model has taken attention to learn more about TH function during development. Here, Lazcano et al. generated crispants for all the TR to show that TH signaling participates in early zebrafish development and to identify TR isoform-specific mediated regulation of early gene expression. Birds also provided their contribution thus highlighting its position as a formidable vertebrate model to study maternal thyroid function (Darras). The data presently available clearly indicate that maternal derived THs play an important role in avian embryonic neurodevelopment linked to post hatch fitness. As a whole, this works highlights the conserved role of maternal thyroid hormones in vertebrate neurodevelopment.

There is no doubt that fine-tuning of TH signaling is essential for proper vertebrate development. Unfortunately, aquatic and terrestrial environments are increasingly contaminated by anthropogenic sources that have the potential to disrupt endocrine function, including TH action. Again, anuran postembryonic metamorphosis provides a powerful model to serve as a sensitive test for the detection and mechanistic elucidation of TH disrupting activities of chemical contaminants and their complex mixtures. As reviewed by Thambirajah et al., sensitive assays are indispensable for the timely detection of TH disruption. Thus, providing a comprehensive understanding of how TH signaling is disrupted with building of adverse outcome pathway is required to isolate molecular dysfunction that precedes adverse effects and complement morphological, behavioral, and histological assessments. In the same context, Leemans et al. focuses on plant protection products, because some pesticides and biocides were recognized as potential TH signaling disrupting chemicals particularly during pre- and perinatal development, two vulnerable periods of exposure. Finally, both reviews highlight the absence from regulatory directives of thyroid-specific endpoints for neurodevelopmental effects.

With this Research Topic, we provide a developmental as well as evolutionary picture to how TH action become so important in vertebrate development. There is no doubt that, actual research on TH role in invertebrates, vertebrate such as amphioxus and lamprey, fishes, amphibians, turtles/reptiles, birds and mammals will provide pertinent results to fully understand the evolution of TH signaling pathway in order to better characterize and treat TH associated pathologies or anthropomorphic disruption of TH signaling.

## Author Contributions

Both authors contributed equally to the manuscript.

### Conflict of Interest

The authors declare that the research was conducted in the absence of any commercial or financial relationships that could be construed as a potential conflict of interest.

